# Use of Zoo Mice in Study of Lymphocytic Choriomeningitis Mammarenavirus, Germany (Response)

**DOI:** 10.3201/eid3001.231521

**Published:** 2024-01

**Authors:** Calvin Mehl, Claudia Wylezich, Christina Geiger, Nicole Schauerte, Kerstin Mätz-Rensing, Anne Nesseler, Dirk Höper, Miriam Linnenbrink, Martin Beer, Gerald Heckel, Rainer G. Ulrich

**Affiliations:** Friedrich-Loeffler-Institut, Greifswald–Insel Riems, Germany (C. Mehl, C. Wylezich, D. Höper, M. Beer, R.G. Ulrich);; German Center for Infection Research, Hamburg–Lübeck–Borstel–Riems, Germany (C. Mehl, R.G. Ulrich);; Zoo Frankfurt, Frankfurt, Germany (C. Geiger, N. Schauerte);; German Primate Center, Leibniz Institute for Primate Research, Göttingen, Germany (K. Mätz-Rensing);; Landeslabor Hessen, Giessen, Germany (A. Nesseler);; Max Planck Institute for Evolutionary Biology, Plön, Germany (M. Linnenbrink);; University of Bern, Institute of Ecology and Evolution, Bern, Switzerland (G. Heckel)

**Keywords:** lymphocytic choriomeningitis virus, host, virus lineage, western Germany, *Mus musculus domesticus*, zoo, house mouse, primate

**In Response:** Gouy de Bellocq et al. question in their letter whether data from zoos can be used to test a biogeographic hypothesis regarding lymphocytic choriomeningitis mammarenavirus (LCMV) (1). We agree that this should be done with caution because zoos may act as hubs for pathogen transfer through captive animal transfer and the use of feeder rodents. As we stated in our article (*2*), the occurrence of LCMV in house mice in western Germany was already described in the 1960s, although genetic information is not available (*3*). The detection of LCMV lineage I in house mice from this zoo and the previous detection of a closely related strain in another zoo in this part of Germany (*4*) is in line with a biogeographic pattern.

We note that we made no claims toward the biogeography of LCMV lineages or of the wild house mice in the zoo. Rather, the study provided multiple evidence that did not support the subspecies host specificity because both LCMV lineages were found in the same population of wild *Mus musculus domesticus* mice in the zoo. The high similarity between LCMV genome sequences from a primate and a wild house mouse suggests a transmission link between captive and wild animals in the zoo. The primate was born in the zoo, and the zoo did not breed mice and has not fed mice to primates for decades; thus, the route through which LCMV might have entered the zoo remains unknown. More detailed analyses will be necessary to test the association of LCMV lineages with their reservoir hosts. The scarcity of LCMV detection in wild rodent populations and pet rodents (*5*) and the co-detection of both LCMV lineages (*2*,*6*) will continue to pose a challenge to biogeographic hypothesis testing.
